# HIV-1 drug resistance-associated mutations among HIV-1 infected drug-naïve antenatal clinic attendees in rural Kenya

**DOI:** 10.1186/1471-2334-13-517

**Published:** 2013-11-04

**Authors:** Michael Kiptoo, James Brooks, Raphael W Lihana, Paul Sandstrom, Zipporah Ng’ang’a, Joyceline Kinyua, Nancy Lagat, Fredrick Okoth, Elijah M Songok

**Affiliations:** 1 Centre for Virus Research, Kenya Medical Research Institute, Nairobi, Kenya; 2 National HIV & Retrovirology laboratories, Public Health Agency of Canada, Ottawa, Ontario, Canada; 3 Institute of Tropical Medicine and Infectious Disease, Jomo Kenyatta University of Agriculture and Technology, Nairobi, Kenya; 4 Department of Medical Microbiology and Infectious Diseases, University of Manitoba, Manitoba, Canada

## Abstract

**Background:**

Access to antiretroviral therapy (ART) has increased dramatically in Sub-Saharan Africa. In Kenya, 560,000 people had access to ART by the end of 2011. This scaling up of ART has raised challenges to the Kenyan health system due to emergence of drug resistant viruses among those on treatment and possible onward transmission. To counter this, and come up with an effective treatment strategy, it has become vital to determine baseline mutations associated with drug resistance among the circulating strains of HIV-1in Kenya.

**Methods:**

The prevalence of mutations associated with drug resistance in HIV-1 protease (PR) and reverse transcriptase (RT) regions from 188 HIV-1 infected treatment-naïve pregnant women was investigated in Kapsabet, Nandi Hills and Kitale district hospitals of Kenya. Blood samples were collected between April 2005 and June 2006. The HIV-1 pol gene was amplified using primers for HIV-1 PR and RT and sequenced using the BigDye chemistry. The mutations were analyzed based on the IAS algorithm as well as the Stanford University HIV Drug Resistance Database.

**Results:**

Based on the PR and RT sequences, HIV-1 subtypes A1 (n=117, 62.2%), A2 (n=2, 1.1%), D (n=27, 14.4%), C (n=13, 6.9%), G (n=3, 1.6%), and possible recombinants (n=26, 13.8%) were detected. Mutations associated with nucleoside reverse transcriptase inhibitors (NRTI) and non-nucleoside RTI (NNRTI)-resistance were detected in 1.6% (3 of 188) and 1.1% (2 of 188), respectively. Mutations associated with PI resistance were detected in 0.5% (1 of 188) of the study population.

**Conclusion:**

The prevalence of drug resistance among drug-naïve pregnant women in rural North Rift, Kenya in 2006 was 3.2%. Major drug resistance mutations associated with PIs, NRTIs and NNRTIs do exist among treatment-naïve pregnant women in North Rift, Kenya. There is a need for consistent follow-up of drug-naïve individuals in this region to determine the impact of mutations on treatment outcomes.

## Background

In Kenya, the first AIDS case was recognized in 1984 [1] and since that time, HIV/AIDS still remains a huge barrier to social and economic development. Currently, the overall country prevalence is estimated to be 6.3% for Kenyans aged 15–49 years. About 1.5 million Kenyan adults are living with HIV. One of the major responses to the HIV/AIDS crisis in Kenya has been the introduction of a national policy for infected persons to access antiretroviral therapy (ART). To implement this policy, the Kenya National ARV program was established in 2002 to progressively deliver effective ARV, reaching 20% of patients by 2005 and 50–60% by 2008. According to World Health Organization (WHO) and Kenya National AIDS and STI Control Programme (NASCOP), >60% of adult Kenyans who were living with HIV and in need of ART were receiving it by the end of 2011 [2].

Gaining access to ART has implications in improving the quality of life for people living with HIV/AIDS and prevention of vertical transmission [3,4]. One of the major obstacles in achieving the long-term efficacy of ART is the development of resistance. In resource-limited settings, adherence is a major contributing factor to development of drug resistance and ultimately treatment failure [5].

Studies on drug resistance have been carried out extensively on HIV-1 clade B viruses, the prevalent HIV-1 subtype in the United States and Western Europe. Majority of HIV strains in developing countries are non-Subtype B. There is hence less data on resistance patterns in such settings. There are reports that non-B HIV-1 strains carry *pol* gene polymorphisms that may result in lower susceptibility to ART compounds than in subtype B [6]. In Kenya, several studies have been done to document drug resistance among drug-naïve [7,8] as well as drug experienced HIV-1 infected individuals [9]. But to date, scanty data is available on the prevalence of drug resistance among HIV-1-infected drug-naïve antenatal clinic attendees in rural settings. It has been shown that transmitted drug resistance mutations impact negatively on first-line ART outcomes [9]. This study was therefore carried out to elucidate the patterns of drug resistance-associated mutations among drug-naïve antenatal clinic attendees in Kenya, especially with the rapid expansion of ART.

## Methods

### Study subjects and specimens

From April 2005 to July 2006, 298 HIV-positive women attending antenatal clinics in Nandi Hills, Kapsabet, and Kitale hospitals were included in this study. After informed consent, participants’ demographic data was obtained using a structured questionnaire. Prior exposure to ART and specifically single dose nevirapine was also explored. The study participants have been previously described [10]. Routine investigations in Kenyan antenatal clinics constitute blood withdrawal for HIV testing.

From each mother, five milliliters of venous blood was collected in sterile vacutainer tubes containing EDTA as anticoagulant. The blood was separated into plasma and buffy coats within six hours by centrifugation. Two aliquots of each sample were immediately kept at -80°C. The samples were later transported under refrigeration (with ice packs) by overnight courier to the laboratory for analysis.

This study was approved by the Kenya Medical Research Institute Scientific Steering Committee and Ethical Review Board. The study was conducted according to the national and international regulations governing the use of human subjects in biomedical research.

### HIV-1 serology

HIV antibodies were tested by rapid tests as per the guidelines laid down by the Ministry of Health, Kenya. Rapid parallel testing was carried out using Determine™ HIV-1/2 (Abbott Diagnostic Division) and Uni-Gold™ HIV (Trinity Biotech) test kits. In case of discrepancy, Bioline HIV 1/2 3.0 (Standard Diagnostics) was used as a tiebreaker.

### Extraction of nucleic acids, polymerase chain reaction (PCR) and sequencing

HIV-1 RNA was extracted from 200 μl of plasma using BioMérieux EasyMAG® robot. An initial One-Step RT-PCR for the *pol* (PR codons 1–99 and RT codons 1–237) region was performed using the Qiagen One-Step RT PCR Kit followed by a nested PCR amplification using in-house group M consensus primers as previously described [9,11].

PCR products were purified using MultiScreen Separations System. Amplicons were sequenced employing BigDye chemistry (Applied Biosytems, Foster City, CA, USA) using in-house primers on an ABI Prism 3130XL sequencer (Applied Biosystems, Foster City, CA, USA). Generated sequences were assembled using Seqscape v2.5 (Applied Biosystems). The resulting sequences were edited using sequence alignment editor software BioEdit v7.0.5 (Ibis Therapeutics, Carlsbad, CA).

### Drug resistance analysis

The edited sequences were analyzed using the WHO’s list of drug resistance mutations for surveillance of transmitted HIV-1 drug-resistance [12]. This analysis list contains 93 mutations of which 34 are NRTI-associated resistance mutations at 15 RT positions, 19 are NNRTI-associated resistance mutations at 10 RT positions and 40 PI-associated resistance mutations at 18 protease positions. The Stanford University HIV Drug Resistance Database (http://sierra2.stanford.edu/sierra/servlet/JSierra) was initially used to determine possible mutations. The mutation points associated with drug resistance were then documented.

### HIV subtyping

Multiple alignments of the generated sequences with reference sequences from the HIV sequence database (Los Alamos National Laboratory) were performed using Clustal W [13]. Genetic distances were calculated by the Kimura two-parameter method [14] and the phylogenetic tree constructed by the neighbor-joining method [15] with its reliability being estimated by 1000 bootstrap replications. The REGA HIV-1 subtyping tool (http://hivdb.stanford.edu/RegaSubtyping/) was used to determine the HIV-1 subtype alongside the HIV drug resistance on the Stanford University HIV Drug Resistance Database (http://hivdb.stanford.edu/).

## Results

### Study samples

Of the 298 plasma samples available, 44 plasma samples did not amplify due to possible low viral load or primer mismatches; 37 were excluded because they were obtained from mothers who had been exposed to single dose nevirapine. Two hundred and fifty four samples were successfully amplified. However, 29 samples did not yield the desired sequence length and were therefore excluded from the final analysis. A final total of 188 HIV-1 *pol* gene sequences were successfully obtained from blood samples of drug-naïve antenatal clinic attendees; 65 from Nandi hills, 71 from Kitale, and 52 from Kapsabet.

### HIV-1 subtype distribution

All the 188 sequences were identified as belonging to HIV-1 group M. They were classified into the following subtypes and circulating recombinant forms (CRFs): subtypes A1 (n=117, 62.2%), A2(n=2, 1.1%), D (n=27, 14.4%), C (n=13, 6.9%), G (n = 3, 1.6%), and possible recombinants (n=26, 13.8%). The possible recombinants included AC (n=3, 1.6%), AD (n=20, 10.6%), CD (n=2, 1.1%), CRF10_CD (n = 1, 0.5%). Distribution of the HIV-1 subtypes in the three hospitals is summarized in Table 1.

**Table 1:** Distribution of HIV-1 subtypes among HIV positive drug naïve Antenatal clinic attendees in rural Kenya

**Subtype**	**Hospital/Facility**			**Total**
	**SNH n=65**	**KAPH n=52**	**KTL n=71**	
**A1**	39	31	47	117
**A2**	1	0	1	2
**C**	4	2	7	13
**D**	13	10	4	27
**G**	1	1	1	3
**AC**	1	0	2	3
**AD**	5	7	8	20
**CD**	1	0	1	2
**CRF 10_CD**	0	1	0	1

SNH: South Nandi Hospital; KAPH: Kapsabet hospital; KTL: Kitale.

### Protease inhibitor (PI) resistance-associated mutations

A major protease inhibitor-associated mutation was found in one strain. This occurred at amino acid position 46 in the HIV protease gene (i.e. M46L). Other major protease inhibitor-associated mutations (D30N, V32I, I47A/V, G48V, I50L/V, I54L/M, L76V, V82A/F/L/S/T, I84V, and L90M) were not detected among the women from the three hospitals. However, minor PI associated mutations detected included L10I (12), L10V (10), L10I/V (3), L33F (1), and L33I (1). Thus all the patients were susceptible to available protease inhibitor regimens.

### Reverse transcriptase inhibitor (RTI) resistance-associated mutations

Primary mutations associated with nucleoside RTI (NRTI), K65R, D67N and K70E were detected in one case each. A secondary mutation, T69S was detected in three women. A mutations associated with non-nucleoside RTI (NNRTI), K101E was detected in one case. Overall, 3.2% of the blood samples from antenatal clinic attendees of the three hospitals harbored drug resistance mutations.

## Discussion

In this study we showed a high diversity of HIV-1 subtypes circulating in the north rift valley region of Kenya. It has been shown previously that HIV-1 diversity is high in Kenya, with a dominance of subtype A1 [7,16]. Similarly, majority of the mothers in this study were infected with HIV-1 subtype A1. Although consistent with other findings in which subtype A1 has been reported to be the major subtype in circulation, the percentage of subtype A1 in this population was lower than what was found in another independent study along the coastal region of Kenya [16]. Nonetheless, there appear to be more recombinants in this population suggesting possible evolution and diversification of prevalent subtypes. We have further shown that the prevalence of drug resistance among drug naïve antenatal clinic attendees in north rift valley in 2006 was 3.2%. This was below the WHO threshold of 5%.

As in other cross-sectional studies, this study had limitations. One, the samples were anonymously collected with no baseline information regarding demographics, viral load or CD4 data that would have enabled us to further categorize the patients for better management later. Two, only positive samples were analyzed for subtypes and drug resistance; site prevalence analysis of HIV in the studied population was not possible. Three, at the time of sample analysis, the ART coverage in Kenya was <20%. The coverage today is > 60%. The case scenario at that time may not be reflective of current status of transmitted resistance. Four, patients in this study did not meet the WHO standard for surveillance drug resistance; it would be hard to establish a definite result on transmitted resistance mutations in such patients with chronic HIV infection. In our setting the study may have underestimated the rate of transmission in this part of Kenya. However, this points to the need for rapid surveillance of resistance among drug naïve individuals in Kenya. All notwithstanding, this study may be a representative of the situation on ART naïve individuals and prevalent subtypes in this region at that time. From our observation, it is likely and possibly true that ART in this population had not gained much ground. Furthermore, if there might have been transmitted mutations, the population sequencing methodology that we employed might have left out mutations that occur as minor populations.

This study provides the first results on potential resistance of HIV-1 to antiretroviral drugs among untreated pregnant women in Kenya. The low frequency of drug resistance mutations in this study is similar with previous studies conducted among drug naïve antenatal clinic attendees in countries close to Kenya, such as Tanzania and Ethiopia [17,18].

## Conclusion

As seen from this study, continuous HIV-1 characterization and evaluation of drug resistance before and during HIV therapy among antenatal clinic attendees in rural set ups would be important in guiding patient directed treatment. This will eventually help to avert the envisaged treatment failure among patients who are increasingly presenting with diverse viral subtypes with limited ART options.

### Sequence data

Sequences obtained in this study were deposited in the GenBank under accession numbers: KC516873 - KC517060.

## Competing interest

The authors declare that there are no conflicts of interest.

## Authors' contributions

MK conceived the study, carried out field work, sample analysis and manuscript preparation; RL carried out sample analysis and manuscript preparation, ZN coordinated field work, sample collection, overall supervision and manuscript preparation; JB and PS carried out molecular analysis; JK and NL carried out sample preparation and amplification; FO and EMS did project implementation, oversight and manuscript preparation. All authors read and approved the manuscript.

## Pre-publication history

The pre-publication history for this paper can be accessed here:

http://www.biomedcentral.com/1471-2334/13/517/prepub
